# Sensory Transduction in Photoreceptors and Olfactory Sensory Neurons: Common Features and Distinct Characteristics

**DOI:** 10.3389/fncel.2021.761416

**Published:** 2021-10-08

**Authors:** Federica Genovese, Johannes Reisert, Vladimir J. Kefalov

**Affiliations:** ^1^Monell Chemical Senses Center, Philadelphia, PA, United States; ^2^Department of Ophthalmology, Gavin Herbert Eye Institute, University of California, Irvine, Irvine, CA, United States; ^3^Department of Physiology and Biophysics, University of California, Irvine, Irvine, CA, United States

**Keywords:** photoreceptor, rod, cone, phototransduction, olfaction, olfactory sensory neuron, vision, signal transduction

## Abstract

The past decades have seen tremendous progress in our understanding of the function of photoreceptors and olfactory sensory neurons, uncovering the mechanisms that determine their properties and, ultimately, our ability to see and smell. This progress has been driven to a large degree by the powerful combination of physiological experimental tools and genetic manipulations, which has enabled us to identify the main molecular players in the transduction cascades of these sensory neurons, how their properties affect the detection and discrimination of stimuli, and how diseases affect our senses of vision and smell. This review summarizes some of the common and unique features of photoreceptors and olfactory sensory neurons that make these cells so exciting to study.

## Introduction

Photoreceptors and olfactory sensory neurons (OSNs) have highly specialized structures that enable them to capture their respective stimuli of light and odorant ligands. Both photoreceptors and OSNs have evolved highly specific abilities to detect and discriminate light wavelengths or odors. They use intricate transduction mechanisms to convert sensory stimuli into electrical signals. Their transduction cascades not only are able to greatly amplify the signal but also to enhance the signal to noise, enabling these cells to detect and distinguish minute stimuli within very noisy backgrounds conditions. Such transduction mechanisms provide for modulation at multiple steps to adapt the sensory neurons to different background stimulation and optimize the capture of useful information about the surrounding world.

In this review, we summarize some of the key structural and functional features of vertebrate rod and cone photoreceptors and of OSNs, and the molecular mechanisms that underlie their function. While describing features of both cell types, we emphasize the similarities and differences between photoreceptors and OSNs and the unique features of each cell type that make them perfectly suited to perform their function.

### Signal Detection in Photoreceptors and Olfactory Sensory Neurons’ Specialized Cilia

**Figure 1:**
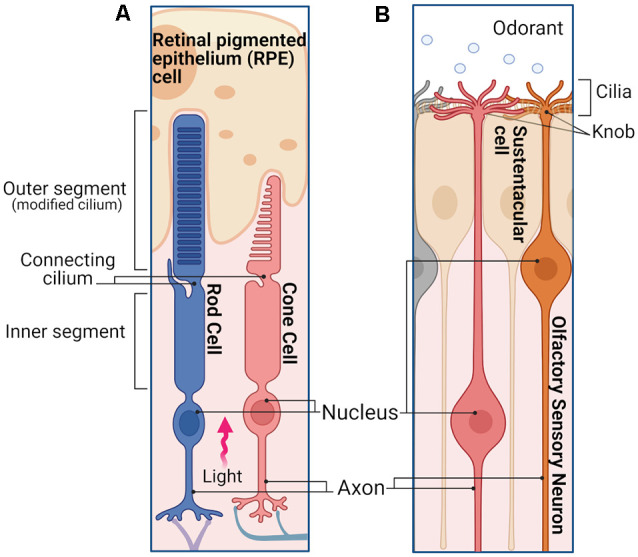
Photoreceptors and olfactory sensory neurons (OSNs).** (A)** Simplified schematic representation of a rod and a cone in the retina. Photoreceptors are polarized neurons with a specialized morphology optimized to detect light stimuli. The outer segments of both rods and cones are modified sensory cilia, containing membrane disks organized in a stack. In the case of rods, the outer segment has a slim rod-like structure in which the disks are enclosed by the plasma membrane. The outer segment of the cones has a stocky conical-shaped structure, in which the disks are constituted by invaginations of the plasma membrane. The outer segment does not contain any proteins of the cell translation machinery, which are mostly localized in the inner segment, including the endoplasmic reticulum, Golgi, and mitochondria. Outer and inner segments are connected by the connecting cilium, while distal to the inner segment is the cell body containing the nucleus, followed by the axon and synaptic termini that extend into the outer plexiform layer where they synapse with the second order neurons. When the light enters the eye, after reaching the retina, it travels along the length of the rod and cone inner segment until finally reaching the outer segments. **(B)** Simplified schematic of an OSNs in the olfactory epithelium. OSNs are ciliated bipolar neurons, their apical dendrites extend to the surface of the epithelium terminating with a spherical structure called dendritic knob, from which the sensory cilia enter the mucus layer. The ciliary membrane contains the olfactory receptors (ORs) necessary to detect different odorants. Distal from the knob is the cell body of the OSN with its nucleus, followed by a long axon that projects to the olfactory bulb, where it synapses with the second order neurons. Images created with BioRender.com.

Vertebrate rod and cone photoreceptors as well as OSNs are ciliary neurons (Figure 1) with specialized cilia where the initial detection of the sensory stimulus takes place to activate a sensory transduction cascade. Rods and cones have a single cilium that has evolved to accommodate a stack of ~1,000 membrane disks where the visual pigment is expressed at a very high 3–5 mM concentration (Figure 1A; Palczewski, 2006). In the case of rods, the disks are enveloped by the plasma membrane, whereas in cones the disks are formed by invaginations of the plasma membrane. As light enters the eye and reaches the retina, it travels along the length of the rod and cone outer segments. The orientation of the elongated outer segments along the light path, together with the high density of visual pigment in their disks results in ~50% probability that an incident photon is absorbed by a visual pigment molecule (Bowmaker and Dartnall, 1980). In the case of OSNs (Figure 1B), odorant ligands are detected in the ~20 cilia protruding from each dendritic knob which are immersed in the mucus layer covering the olfactory epithelium. The olfactory cilia, which are motile in amphibians but not in rodents, are only about 0.1–0.2 μm thin but can reach up to 100 μm in length depending on the species (Kleene and Gesteland, 1981; Ukhanov et al., 2021). While this greatly increases the surface membrane area available to incorporate olfactory receptor (OR) proteins to detect odorants, it also greatly reduces the ciliary volume with potentially detrimental effects (see below).

### Electrophysiological Approaches to Record Light- and Odorant-Induced Responses

**Figure 2:**
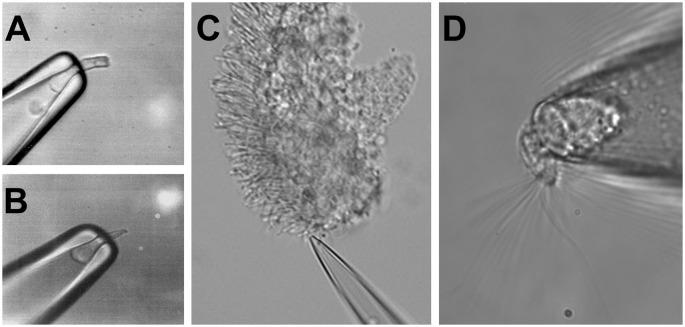
Suction electrode recordings of photoreceptors and OSNs. Suction electrode with a sucked salamander rod **(A)** and a salamander cone **(B)**. **(C)** Suction electrode with a mouse rod from a fragment of the dissected retina. **(D)** Suction electrode with a single salamander OSN. Figures modified from Reisert and Matthews (2001), Kefalov et al. (2010), and Kefalov (2012) with permission.

The similar morphological structure of rods, cones, and OSNs, with a ciliary part able to detect the respective stimuli and an adjacent cell body, allows similar electrophysiological approaches to record stimulus-induced responses in these cell types. The cell body of a photoreceptor or an OSN can be sucked into the tip of a recording pipette by using a loose-patch (or suction pipette) recording configuration (Baylor et al., 1979; Lowe and Gold, 1991). This leaves the outer segment of photoreceptors or the olfactory cilia exposed and accessible to bath solution changes, e.g., the application of pharmacological agents or to odorants, in the case of OSNs. Suction pipette recordings can be performed from isolated sensory neurons, as shown in Figures 2A,B,D (respectively, a salamander rod, salamander cone, and salamander OSN) but also from dissected retina tissue, as in the case of the outer segment of a mouse rod drawn in the recording electrode from a piece of the retina (Figure 2C). This recording configuration measures the transduction current entering the photoreceptor outer segment or olfactory cilia, and leaving *via* the cell body.

**Figure 3:**
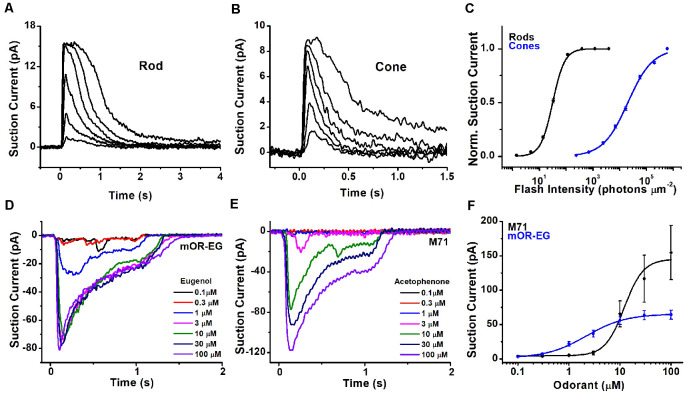
Light and odorant-induced responses. Typical response families from single-cell suction current recordings obtained from a mouse rod **(A)** and cone **(B)**. 10 ms flashes of various intensities were delivered to each photoreceptor at time 0, with each successive flash 0.5-log times brighter than the previous one. **(C)** Normalized intensity-response functions showing that cones have approximately 2.5 log units lower sensitivity in comparison to rods. Suction current recordings from OSNs expressing the mOR-EG **(D)** or M71 **(E)** olfactory receptor, stimulated with increasing concentrations of the odorants eugenol and acetophenone, respectively. **(F)** Dose-response curves showing different sensitivity of mOR-EG and M71-expressing OSNs. Figures modified from Kefalov et al. (2010), Sakurai et al. (2011), Deng et al. (2013), and Dibattista and Reisert (2016) with permission.

A fundamental difference between photoreceptors’ and OSNs’ responses to stimuli lies in their polarity. In the absence of light, rods and cones are kept depolarized by a standing inward current of approximately 20–40 pA for amphibian cones and rods, and 7–15 pA for mouse photoreceptors. This depolarizing current is gradually suppressed upon light stimulation until, for sufficiently high light intensities, it is reduced to zero (Figures 3A,B, mouse rod and cone responses, respectively), leading to photoreceptor hyperpolarization. Similar to rods, but differently from cones, the OSNs show comparatively little spontaneous activity in absence of stimuli (Reisert, 2010; Connelly et al., 2013). Different OSNs show varying levels of spontaneous basal activity determined by the constitutive activity of their ORs (Reisert, 2010; Connelly et al., 2013).

In the presence of odorants, OSNs generate an inward receptor current which leads to depolarization, and the generation of action potentials (Firestein and Werblin, 1989; Kurahashi, 1989; Reisert and Matthews, 1999). This receptor current is odorant concentration-depend and increases progressively with increasing stimulation until it eventually saturates at high odorant concentrations. Responses recorded from OSNs expressing different olfactory receptors can generate fairly different response amplitudes when stimulated with their respective agonists (Figures 3D,E: responses recorded from mouse OSNs that express the mOR-EG or the M71 olfactory receptor, which are activated by the ligands eugenol and acetophenone, respectively).

The hyperpolarization and signals carried by graded potentials in photoreceptors vs. depolarization and signals carried by action potentials in OSNs represent another fundamental difference between these two types of sensory neurons. These topics and the differences in synaptic structure and transmission between photoreceptors and OSNs go beyond the focus of this review and are discussed in an excellent recent review on this topic (Lankford et al., 2020).

### Sensitivity of Photoreceptors and Olfactory Sensory Neurons

In part due to their unique structure, photoreceptors, and, to a lesser extent, OSNs have achieved exquisite sensitivity that optimizes the detection of stimuli within the respective sensory organs. In addition, both sensory receptors use a transduction cascade to amplify the signal (see below). As a result, rod photoreceptors can reliably detect single photons (Baylor et al., 1979), enabling humans to perceive light with as few as six photons detected by adjacent rods (Hecht et al., 1942). This renders rods perfectly suited for dim light vision, with a dynamic range spanning lights from a dark cloudy night to sunrise (Fain et al., 2010). Cones are ~100-fold less sensitive than rods, making them suited for daytime light conditions. Figure 3C compares the intensity-response function of mouse rods and cones, demonstrating the much lower sensitivity of cones compared to rods.

Most OSNs respond to odor concentrations in the low micromolar range (Bozza et al., 2002; Grosmaitre et al., 2009; Saito et al., 2009; Lee et al., 2011; Dibattista and Reisert, 2016), but they can also reach exquisite sensitivity and are capable of detecting odors at the nanomolar concentration range. Picomolar sensitivity is reached by a subset of OSNs that express receptors specialized in detecting amines, the trace-amine associated receptors (Zhang et al., 2013). In comparison to rods, OSNs do not reach such high sensitivity, and cannot be activated by a single odorant molecule but instead require around 30 odorant binding events to begin firing action potentials reliably (Bhandawat et al., 2010). The detection of odorants in the olfactory epithelium can be further enhanced by the expression of a wider number of different OR genes, more than 350 in humans and 1,000 in mice (Malnic, 2007), with overlapping response profiles to odorants. A larger number of OSNs, particularly in species relying heavily on their sense of smell, may enhance further the detection of odorants. For instance, the human olfactory epithelium covers ~3–4 cm^2^ and contains approximately 5–6 million OSNs while in the case of dogs, the area of the olfactory epithelium is 18–150 cm^2^ and contains 150–300 million OSNs (Lippi and Heaney, 2020).

### Detection of Stimuli

In both photoreceptors and OSNs, the detection of stimuli is mediated by G protein-coupled receptors. In photoreceptors, this function is achieved by rod and cone visual pigments, which consist of a protein, opsin, covalently attached to the visual chromophore, typically 11-cis-retinal (Ebrey and Koutalos, 2001). The chromophore serves as a reverse agonist, keeping the receptor molecule in the inactive ground state (Crouch et al., 1996). Absorption of a photon by 11-cis-retinal triggers its conformational change to all-trans-retinal, which, in turn, results in rearrangement of the opsin transmembrane helices and switch of the visual pigment molecule into its active state. The activated visual pigment then binds to a G protein, transducin, activating it. The activation of transducin triggers the transduction cascade that ultimately generates the cellular response (Pugh and Lamb, 1993). Eventually, the all-trans retinal chromophore is released from opsin after the covalent Schiff base between them is hydrolyzed, leaving behind chromophore-free opsin (Saari, 2016). Notably, without chromophore, opsin has residual activity, and in sufficient quantities can produce steady activation of the photoreceptors, similar to a steady background light, thus modulating the sensitivity of photoreceptors (Fain et al., 1996). This process is known as bleaching adaptation, indicating the production of free opsin after the photoactivation of the visual pigment and dissociation of the visual chromophore.

Unlike in photoreceptors, where the ligand, a light-sensitive reverse agonist, is covalently attached to opsin, in olfaction, the ligands are dissolved in the mucus covering the surface of the olfactory epithelium and come into direct contact with the OR proteins expressed in the OSN ciliary membrane. This results in the activation of the receptor protein that, in turn, is transduced downstream to a G protein to trigger a transduction cascade resulting in the cellular response. The binding of the ligand to the receptor protein is noncovalent and rapidly reversible. ORs, like other G protein-coupled receptors, do display antagonism, inverse, and partial agonism, leading to suppressed responses to their agonists, a reduction in basal activity in the absence of stimulation or suppression of the maximal response (Firestein et al., 1993; Oka et al., 2004; Reisert, 2010).

### Discrimination Between Stimuli

The spectral sensitivity of individual rod and cone photoreceptors is dictated by the absorption properties of their visual pigments. Typically, each photoreceptor type expresses only one type of opsin; in the case of the human retina, rods express rod opsin, whereas cones express long wavelength (LW, red), middle wavelength (MW, green), or short wavelength (SW, blue) opsin (Nathans, 1987). When bound to the chromophore, the amino acid structure of each opsin determines the optical properties of the resulting visual pigment and the spectral sensitivity of the photoreceptors expressing it. As a result, species existing in environments with characteristic light distribution, such as deep-sea fish, have visual pigments that have evolved to optimize their spectral sensitivity (Hope et al., 1997). A second factor controlling the optical properties of the visual pigment is the structure of the visual chromophore. Most species, including mice and humans, use 11-cis-retinal, a derivative of Vitamin A, also known as A1. However, some amphibians and fish also use 3,4-dehydro 11-cis retinal, also known as A2. This chromophore has an extra conjugated double bond in its structure, which shifts the absorption spectrum of A2 visual pigments to longer wavelengths compared to A1 visual pigment embedded in the same opsin molecule (Corbo, 2021). Some aquatic and amphibian species use the A1/A2 chromophores to shift their spectral sensitivity from murky waters dominated by longer wavelengths of light to seawater and air, dominated by shorter wavelength lights (Bridges, 1964). One notable example includes the toad, where the retina is populated by A1 visual pigment in its ventral section, receiving light from above the surface of the water, and by A2 visual pigment in its dorsal section, receiving light from below the surface of the water (Reuter et al., 1971). A shift in the chromophore can also occur during the lifetime of the animal as its environment changes, such as the A2 to A1 shift in salamanders as they metamorphose from larval (aquatic) to the adult (terrestrial) stage (Ala-Laurila et al., 2007), or the A2 to A1 shift in Atlantic salmon during migration from sea to freshwater (Beatty, 1966).

Similar to photoreceptors, the ligand specificity of the OSNs is also dictated by the expression of OR genes in their cilia. As photoreceptors, each OSN expresses generally only one receptor gene so that its ligand specificity is determined by the structure of the OR expressed in that particular cell. However, photoreceptors typically use no more than five opsin genes to cover the visible spectrum, while OSNs can use hundreds, in the case of humans, to thousand and more, for rodents and dogs, OR genes to cover the odor space (Malnic, 2007). The same OR can be activated by multiple odorants with different sensitivities, and a given odorant can activate different ORs with different half-maximal concentrations (Buck, 1996; Ache, 2020). This generates a complex mosaic of ORs and odorants response pairs. Figure 3F compares the dose responses of OSNs expressing either the mOR-EG or the M71 OR to eugenol and acetophenone, respectively. In this case, mOR-EG OSNs display higher sensitivity to its agonist compared to M71 OSNs. However, this does not preclude the possibility that the M71 OR is more sensitive to another ligand resulting in a more left-shifted dose response relation than the one seen with acetophenone. Conversely, the dose response relation of M71 OSNs to benzaldehyde is approximately 10-fold right-shifted compared to acetophenone (Bozza et al., 2002).

Determining the ligand specificity of ORs is an ongoing endeavor (Abaffy et al., 2006; Saito et al., 2009; Kurian et al., 2021). Due to the large number and diversity of OR genes, as well as the near endless number of odorant molecules, understanding the overall mechanisms that control their ligand binding affinity and specificity remains a challenge. Receptor modeling approaches to understand and predict OR–odorant molecule interactions can provide valuable insights but are somewhat hampered by the lack of a crystal structure of any vertebrate OR. The rhodopsin structure is often used as a guide and homology model to predict the structure of ORs (Katada et al., 2005; Bavan et al., 2014).

### Sensory Transduction Activation

**Figure 4:**
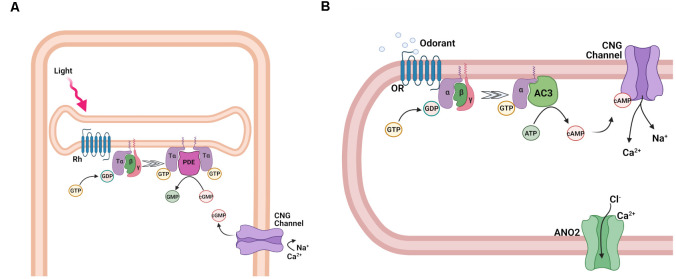
Activation of the transduction cascade in rod photoreceptors and OSNs. **(A)** Schematic representation of phototransduction cascade in rods. Abbreviations: rhodopsin (Rh), Tα, β, and γ subunits of the retinal G protein, transducin (T), guanosine-5′-triphosphate (GTP), guanosine-5′-diphosphate (GDP), phosphodiesterase (PDE), guanosine monophosphate (GMP), and cyclic guanosine monophosphate (cGMP), and cyclic nucleotide-gated (CNG) channel. **(B)** Schematic representation of the olfactory transduction cascade in OSNs. Abbreviations: Olfactory receptor (OR), guanosine-5′-triphosphate (GTP), guanosine-5′-diphosphate (GDP), Gα_olf_, *β*, and *γ*, subunits of the olfactory G protein; adenylyl cyclase 3 (AC3), adenosine-5′-triphosphate (ATP), cyclic adenosine monophosphate (cAMP), cyclic nucleotide-gated (CNG) channel; Ca^2+^-activated Cl^−^ channel anoctamin 2 (ANO2). Images created with BioRender.com.

In both photoreceptors and OSNs, the detection of stimuli by their respective G protein-coupled receptors is converted into electrical signals *via* the activation of a G protein coupled to a second messenger transduction cascade. The two pathways, though clearly distinct, share an amazing level of similarity (Figure 4). Thus, in both cases the second messenger is a cyclic nucleotide, cGMP in photoreceptors (Pugh and Lamb, 1990) and cAMP in OSNs (Sklar et al., 1987; Bakalyar and Reed, 1990). As a result, the activation of both transduction cascades results in a rapid shift in the equilibrium between synthesis and hydrolysis of the respective cyclic nucleotide, which is then sensed by the cyclic nucleotide-gated (CNG) transduction channels in the plasma membrane of the photoreceptor outer segment or olfactory cilium.

In the case of photoreceptors, the photoactivated visual pigment binds to and activates the trimeric G protein transducin (T) (Figure 4A), causing the exchange of GDP for GTP on its α-subunit, which is part of the Gα_t_ protein family. Following the subsequent dissociation of the α-subunit (Tα) from its β/γ complex (Tβγ), Tα then binds to the cGMP phosphodiesterase (PDE) complex, relieving the inhibition of its catalytic α- and β-subunits by its inhibitory γ-subunits (Ebrey and Koutalos, 2001; Burns and Arshavsky, 2005). All these transduction proteins are embedded in or tethered to the disc membranes inside rods or are contained in the cell membrane of cones. As a result of their activation, the hydrolysis of free cGMP in the outer segment is upregulated, causing its rapid decline, and partial or complete closure of the cGMP-gated channels expressed in the rod and cone cell membrane (Luo et al., 2008). The closure of the CNG channels leads to the reduction of the inward transduction current, followed by the hyperpolarization of the cells, and a reduction of neurotransmitter release to second order neurons within the retina. Inversely, in the absence of light, the opening of CNG channels and the resulting inward transduction current is sustained by the continuous cGMP production by guanylyl cyclase (GC).

Similarly, in OSNs (Figure 4B), the ligand-activated OR proteins bind to the G protein G_olf_, causing its dissociation into active Gα_olf_ and olfactory β- and γ-subunit, Gβγ_olf_. In contrast to transducin, however, Gα_olf_ is part of the Gα_s_ protein family and binds to adenylyl cyclase 3 (AC3), activating it. As a result, the synthesis of cAMP in the olfactory cilia is upregulated, causing its rapid increase and the opening of cAMP-gated channels (Kleene, 2008; Su et al., 2009; Boccaccio et al., 2021).

While both photoreceptors and OSNs use CNG transduction channels, their respective channels have different subunit compositions (Bradley et al., 2005). Rods and cones express heterotetramers consisting of the main A1 and A3 and the modulatory B1a and B3 subunits in 3:1 and 2:2 stoichiometries respectively. The olfactory CNG channel is a heterotetramer consisting of two units of the main A2 subunits and one each of the modulatory A4 and B1b subunits. Interestingly, the rod and the olfactory CNG channels express different splice variants of the same B1 subunit. In OSNs, the initial inward Na^+^ and Ca^2+^ current generated by the opening of the CNG channel raises ciliary Ca^2+^ and opens a secondary ion channel, the Ca^2+^-activated Cl^−^ channel Anoctamin 2. A high intraciliary Cl^−^ maintained by the Na^+^/K^+^/2Cl^−^ cotransporter 1 ensures a Cl^−^ efflux which further depolarizes the OSNs (Dibattista et al., 2017; Boccaccio et al., 2021). This depolarization triggers the generation of action potentials which further propagate along the axons, inducing glutamate release at synapses with the second order neurons in the olfactory bulb (Murphy et al., 2004). In photoreceptors, the transduction cascade upon stimulation does not ultimately generate action potentials in the receptor cell, but only a graded receptor potential that directly causes a change in neurotransmitter release.

### Amplification

As for any other sensory modality, proper amplification of the signal is required for the detection of small stimuli and the resulting high sensory sensitivity is critical for the survival and propagation of the species. Nature has reached the highest physically possible sensitivity in the case of rod photoreceptors that can produce a detectable electrical response to the absorption of a single photon. This impressive feat is achieved by employing a transduction cascade that allows tremendous amplification of the signal. During the ~50 ms active lifetime, a single photoactivated rhodopsin molecule activates ~20 transducins, producing an immediate 20-fold amplification (Burns and Pugh, 2010). The following activation of PDE by transducin does not directly produce amplification as each transducin has to bind to a PDE molecule to activate it. However, once activated, each PDE enzyme can hydrolyze thousands of cGMP molecules. Lastly, as the binding of cGMP to the CNG transduction channels is cooperative, a slight change in cGMP levels can reduce the number of cGMP molecules bound to the channel from 3 to 2. This results in channel closure and a sharp reduction in the transduction current, further enhancing the detection of photostimulation. Despite the similarities in the transduction cascades of rods and cones, the amplification in cone photoreceptors is substantially lower as a result of fine-tuning at several of the phototransduction steps (Yau, 1994; Kawamura and Tachibanaki, 2008). Interestingly, even though rod and cone visual pigments activate transducin with similar efficiencies, the lower thermal stability of the cone visual pigment results in higher intrinsic activity in cones compared to rods in darkness (Kefalov et al., 2003), effectively desensitizing the cones and shifting their function towards brighter daytime light conditions (see Figure 3C).

Curiously, the activation of G_olf_ by the OR molecule does not result in amplification. Indeed, the dwell time of the odorant ligand on the OR appears to be very short and on a millisecond timescale (Bhandawat et al., 2005). As a consequence, on average, this results in the activation of less than one G protein per activated receptor. As such, in contrast to phototransduction, where the lifetime of the activated rhodopsin greatly influences the response size and kinetics, in OSNs the response depends more prominently on the coupling efficacy of downstream transduction components while the odorant presence keeps the OR activated. To compensate for the lack of initial amplification at the G protein level, OSNs employ a secondary amplification step on top of the cAMP transduction cascade. The activation of AC3 by G_olf_ results in the synthesis of most likely hundreds of cAMP molecules, the opening of the CNG channels which is followed by a unique secondary amplification based on excitatory Ca^2+^-activated Cl^−^ channels in the cilia (Figure 4B). The Cl^−^ current carries up to 80% of the overall transduction current (Dibattista et al., 2017). Physiological experiments with pharmacological and genetic modulation of the Cl^−^ conductance indicate that the Cl^−^ channels serve to set the length of the action potential train generated in response to odorant stimulation (Pietra et al., 2016) and to promote recognition of novel odorants (Pietra et al., 2016; Neureither et al., 2017).

A puzzling aspect of the secondary amplification step is why Cl^−^ is the charge-carrying ion and not Na^+^, which could be achieved easily by increasing the expression level and/or the ion permeation and conductance of the olfactory CNG channel. Recent theoretical work hinted at two main advantages of Cl^−^, instead of Na^+^, as the charge carrier. As the external environment of cilia is the nasal mucus, currents will depend on the ion concentration in the mucus, which can be unstable. A current that depends on the intracellular ion concentration, as is the case for Cl^−^ but not for Na^+^, is much less dependent on the mucosal ion concentration. For instance, this could become an issue in the case of a cold with a runny nose or during swimming, when the mucus becomes diluted. The second advantage results from the “compromise” to increase the ciliary surface area, at the expense of having a very small ciliary volume, in the femtoliter range. In such small volumes, even small ionic currents can lead to large changes in ion concentration and osmotic pressures. If the main charge carrier was Na^+^ this would lead to a large increase (tens of mM). This would cause a large increase in osmotic pressure and also would prevent Ca^2+^ clearance *via* the olfactory Na^+^/Ca^2+^, K^+^ exchanger (see below) with greatly deleterious effects. In contrast, high intracellular Cl^−^ is maintained throughout the OSN so that its local depletion in the cilia upon ligand activation is rapidly reversed by diffusion from the cell soma. Both these issues do not exist for photoreceptors as they are embedded in the interstitial fluid of the eye and photoreceptors are sufficiently large and their transduction currents are sufficiently small that ion concentration changes due to changes in transduction current are relatively small (Reisert and Reingruber, 2019). Nevertheless, rod photoreceptors undergo osmotically-driven length changes upon light activation, an effect that is mitigated by the translocation of G protein subunits into the cytosol (Zhang et al., 2017).

### Receptor and G Protein Inactivation

**Figure 5:**
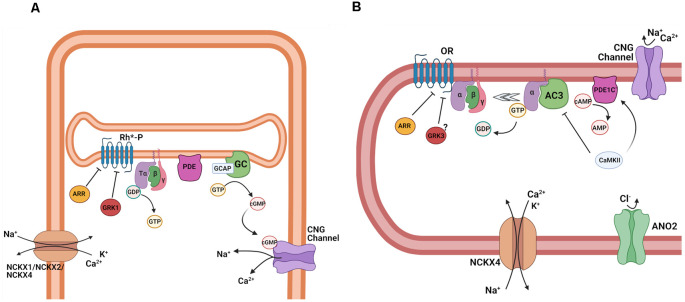
Termination of transduction cascade in rod photoreceptors and OSNs. **(A)** Schematic representation of the termination of phototransduction in rods. Abbreviations: Phosphorylated light-activated rhodopsin (Rh*-P), arrestin (ARR), G protein-coupled receptor kinase 1 (GRK1), Tα, β, and γ subunits of the retinal G protein, transducin (T), guanosine-5′-triphosphate (GTP), guanosine-5′-diphosphate (GDP), phosphodiesterase (PDE), guanosine monophosphate (GMP) and cyclic guanosine monophosphate (cGMP), and cyclic nucleotide-gated (CNG) channel, guanylate cyclase (GC), guanylate cyclase activating protein (GCAP), cyclic nucleotide-gated (CNG) channels, K^+^ dependent Na^+^/Ca^2+^ exchanger 1, 2 and 4 (NCKX1, NCKX2, NCKX4). **(B)** Schematic representation of the termination of the olfactory transduction cascade. Abbreviations: Olfactory receptor (OR), arrestin (ARR), G protein-coupled receptor kinase 3 (GRK3) Gα_olf_, *β*, and *γ*, subunits of the olfactory G protein, guanosine-5′-triphosphate (GTP), guanosine-5′-diphosphate (GDP), adenylyl cyclase 3 (AC3), activated phosphodiesterase 1C (PDE1C), cyclic adenosine monophosphate (cAMP), adenosine monophosphate (AMP), Ca^2+^/calmodulin-dependent protein kinase II (CaMKII), cyclic nucleotide-gated channel (CNG); Ca^2+^-activated Cl^−^channel anoctamin 2 (ANO2), K^+^-dependent Na^+^/Ca^2+^ exchanger 4 (NCKX4). Images created with BioRender.com.

Timely and effective transduction inactivation is critical for allowing sensory neurons to continue to detect stimuli with high temporal resolution. Equally important is to extract behaviorally relevant information from the presented stimuli. In both photoreceptors and OSNs, all active transduction components need to be turned off and the level of cyclic nucleotides within the cells needs to be restored to the rest level before the sensory cell can be reset to the inactive state and become ready for subsequent activation (Figure 5). In the case of photoreceptors, the identity of the step determining the overall kinetics of the photoresponse inactivation was the subject of intense research and debate over several decades. As the visual chromophore ligand is covalently attached to opsin, inactivation of the visual pigment could potentially be extremely slow. Indeed, if left on its own, the active state of rhodopsin decays with a time constant of ~50 s (Imai et al., 2007). Its inactivation in photoreceptors is a two-step process, involving phosphorylation of the rhodopsin C-terminus by rhodopsin kinase (GRK1) which partially quenches its activity, followed by the binding of arrestin1, which completely inactivates the visual pigment (Figure 5A). Though the decay of the active state of cone pigment is significantly faster at ~2 s (Fu et al., 2008), this is still clearly too slow to enable the timely termination and reset of phototransduction. Thus, in both rods and cones, the visual pigments are inactivated by phosphorylation by rhodopsin kinase and the subsequent binding of arrestin long before they would decay spontaneously (Makino et al., 2003). The effective time constant of rod visual pigment inactivation is ~50 ms (Krispel et al., 2006). The slowest step in the inactivation of rod phototransduction turned out to be the hydrolysis of GTP which shuts off Tα, a reaction driven by the transducin GTPase activity and enhanced by a GTPase (GTP-ase activating protein, GAP) complex consisting of Gβ5 and the membrane anchoring protein R9AP (Arshavsky and Wensel, 2013). Inactivation of transducin results in its release from PDE, allowing the two PDE γ inhibitory subunits to resume their inhibition on the two catalytic subunits (α and β) of this enzyme. The kinetics of this reaction determines the overall kinetics of response inactivation in rod photoreceptors. In contrast, work from amphibian cones suggests that in cones the photoresponse duration is Ca^2+^-dependent and involves the quenching of the cone visual pigment (Matthews and Sampath, 2010).

In OSNs, the inactivation by phosphorylation and arrestin are potentially not needed for the timely shutoff of the olfactory transduction cascade, due to the extremely short lifetime of the active ligand-bound receptor molecule. Early biochemical experiments suggested that OR phosphorylation does control cAMP kinetics (Dawson et al., 1993; Schleicher et al., 1993; Peppel et al., 1997), but it seems to play little, if any, role in the control of odorant-response kinetics for one particular OR, mOR-EG (Kato et al., 2014). It still remains to be established whether this applies to all ORs, or whether a subset of ORs is subject to phosphorylation and inactivation. β-arrestin interacts with ORs, mediating internalization during prolonged stimulation and altering adaptation to repetitive odor stimuli (Mashukova et al., 2006). Experiments on isolated human and rat OSNs suggested a role for protein kinases A (PKA) and C (PKC) in the termination of the olfactory response. Ca^2+^ imaging showed that the inhibition of PKA and PKC increases intracellular Ca^2+^ responses in the presence of odorant mixtures, and blocks their termination after odorant stimulation ceases. While the inhibition of both PKA and PKC modulated the odor-induced intracellular Ca^2+^ increase in the human OSNs, only PKC and not PKA affected the Ca^2+^ response to odorants in rat OSNs, suggesting differences among species in the termination of the olfactory response (Gomez et al., 2000).

The control of the lifetime of the olfactory G protein seems to be more complex and less well understood compared to phototransduction. Ric-8B (resistant to inhibitors of cholinesterase-8B) has been identified as a GTP exchange factor (GEF) expressed in OSNs, which facilitates the exchange of GDP for GTP on Gα_olf_ and its activation. Unusually, Ric-8B not only interacts with the G protein α-subunit, but also with γ13, the olfactory γ-subunit. In a heterologous system, Ric-8B co-expression with olfactory transduction components can greatly increase cAMP production, suggesting that it could indeed modulate olfactory transduction (Von Dannecker et al., 2005; Kerr et al., 2008). A knockout mouse for Ric-8B displays impaired olfactory behavior, and, surprisingly, greatly reduced odorant responses. Ric-8B is localized primarily in the cell body and the dendritic knob of OSNs. Ric-8B knockout OSNs are devoid of Gα_olf_ (Machado et al., 2017), suggesting that this gene is needed for the stable expression of Gα_olf_, and excludes addressing its potential role as a GEF in the odorant response. The Ric-8B knockout mice also display higher OSN cell death. Regulators of G protein signaling (RGS) are GAPs that modulate the lifetime of an activated G protein as described above. RGS2, instead of functioning as a GAP, directly inhibits AC3 to control the size of the odorant response (Sinnarajah et al., 2001). However, inconsistent and contradictory data on RGS2 and RGS3 expression and their roles in OSNs suggest that more research is needed (Norlin and Berghard, 2001; Kanageswaran et al., 2015; Saraiva et al., 2015).

### Adaptation

Adaptation plays a critical role in the capacity of our sensory neurons to remain able to detect stimuli above the background in a complex and rapidly changing environment. For instance, in constant light conditions, the dynamic range for both rods and cones is only 100-fold, spanning a range from threshold stimulation to saturation (Figure 3C). However, as a result of light adaptation, photoreceptors can shift their functional range over a very wide span of light conditions, ranging from cloudy night to sunrise for rods, and starry night to bright sunny day for cones (Weale, 1961). Thus, using the adaptation of individual photoreceptors, the visual system is able to remain responsive to stimuli over a wide range of light conditions. In contrast, the ability of OSNs to adapt is rather limited even at modest levels of background odorant (Reisert and Matthews, 1999). Nevertheless, increasing concentrations of the same odorant are able to recruit less sensitive ORs, and therefore less sensitive OSNs, preserving its perception at higher concentrations and ensuring to report the presence of that odorant to the brain.

In both types of sensory neurons, adaptation is mediated by a change in Ca^2+^ upon stimulation. This change is sensed by several Ca^2+^-binding proteins that trigger a negative feedback on the vision and olfaction transduction cascades by modulating several of their steps. In the outer segments of rods and cones and in olfactory cilia, Ca^2+^ levels are controlled by the balance of influx *via* the CNG channels, whose current is carried in part by Ca^2+^, and efflux *via* Na^+^/Ca^2+^, K^+^ exchangers (NCKXs) that use the electrochemical gradient for Na^+^ and K^+^ to extrude Ca^2+^ (Figure 5; Yau and Nakatani, 1984). In rods (Figure 5A), this task is accomplished by the rod-specific NCKX1, whereas cones employ two separate exchangers, NCKX2 and NCKX4 (Vinberg et al., 2017). At rest, both in darkness and in steady state light, the influx of Ca^2+^ is matched with its extrusion and, as a result, the level of free Ca^2+^ in the outer segments is maintained constant. Upon photostimulation, the transduction cascade is activated, resulting in depletion of cGMP, closure of CNG channels, and reduction in the influx of Ca^2+^ into the outer segments. However, Ca^2+^ extrusion by the Na^+^/Ca^2+^, K^+^ exchangers carry on for at least a while and, as a result, the level of Ca^2+^ in the outer segments declines. Direct Ca^2+^ measurements in amphibian photoreceptors indicate a dynamic range from 670 to 30 nM in rods (Sampath et al., 1998) and 400–5 nM in cones (Sampath et al., 1999), in darkness and bright light, respectively.

The light-driven decline in Ca^2+^ causes its release from several Ca^2+^-binding proteins. The dominant Ca^2+^-dependent feedback mechanism in both rods and cones controls the synthesis of cGMP by membrane-bound GC *via* a pair of GC activating proteins (GCAPs)—GCAP1 and GCAP2. When Ca^2+^ in the outer segments is high, Ca^2+^-bound GCAPs bind to and partially inhibit the activity of GC. Upon photoactivation and the decline in Ca^2+^, GCAPs become Ca^2+^-free and release from GC, resulting in upregulation of cGMP synthesis which restores the dark current after photostimulation and modulates the activation of the transduction cascade in the presence of background light (Dizhoor, 2000; Sakurai et al., 2011). Another mechanism by which Ca^2+^ modulates phototransduction involves the Ca^2+^-binding protein recoverin. As GCAPs, recoverin is a member of the EF-hands protein family, and when bound to Ca^2+^ in darkness, it inhibits rhodopsin kinase, thus slowing down the inactivation of the visual pigment (Makino et al., 2004; Sakurai et al., 2015). When the photoreceptors are activated and Ca^2+^ declines, it is released from recoverin, which in turn dissociates from rhodopsin kinase and relieves its inhibition. This enhances the phosphorylation of visual pigments and accelerates their inactivation, effectively reducing the activation of the transduction cascade by the background light. Finally, direct modulation of the CNG channels has also been suggested. However, in the case of rods, such modulation appears to play a marginal, at best, role (Koutalos and Yau, 1996) and is not mediated by the Ca^2+^-binding protein calmodulin (Chen et al., 2010). In zebrafish cones, the modulation of the CNG channels appears to play a more substantial role and is mediated by the Ca^2+^-binding protein CNG-modulin (Korenbrot et al., 2013). It is still unclear whether the mammalian homolog of CNG modulin, EML1 plays a similar role in mammalian cones.

Adaptation in OSNs is less well understood compared to phototransduction. Early data, mostly of biochemical nature or obtained from heterologously-expressed proteins of interest, suggested three main molecular targets for adaptation. All three of them are mediated by the Ca^2+^ influx during the odorant response: Ca^2+^/calmodulin-mediated desensitization of the olfactory CNG channel to close the channel even in the presence of high cAMP (Chen and Yau, 1994); phosphorylation *via* CaM-kinase 2 of AC3 to reduce the rate of cAMP production (Wei et al., 1996, 1998); and Ca^2+^-mediated upregulation of phosphodiesterase 1C, which is expressed in olfactory cilia, and is assumed to degrade cAMP to AMP to terminate the response (Borisy et al., 1992). Follow-up experiments using recordings from OSNs all seem to indicate that none of these mechanisms plays as prominently or as originally thought of role in transduction (Reisert and Zhao, 2011). A mouse with a mutation in the CNGB1b channel subunit that entirely prevents desensitization by Ca^2+^ surprisingly displays normal olfactory adaptation but instead shows a delayed response termination, suggesting that Ca^2+^/calmodulin-mediated desensitization of the CNG channel speeds up response termination (Song et al., 2008). A mouse model that carries a mutation in AC3 that prevents phosphorylation does not show a discernable phenotype of the olfactory response (Cygnar et al., 2012), although it might be possible that other, unknown phosphorylation sites in AC3 might be important. Finally, a knockout mouse for PCE1C has no deficits in response termination but instead shows much reduced response amplitudes for unclear reasons (Cygnar and Zhao, 2009). This begs the obvious question as to what the role of PDE1C might be and what might actually happen to cAMP that is generated during the odorant response. For the latter, an interesting option is that cAMP diffuses out of the cilia into the cell body as a means to reduce ciliary cAMP, allowing OSNs to recover from stimulation (Cygnar and Zhao, 2009). One aspect that is reasonably understood is NCKX4, the Ca^2+^ exchanger in OSNs that is required to lower intraciliary Ca^2+^ during and after odorant stimulation, allowing the transduction cascade to recover from adaptation (Reisert and Matthews, 1998; Stephan et al., 2012).

### Diseases

Disorders affecting photoreceptors are among the leading causes of blindness in the human population. One of the prevalent visual disorders, called retinitis pigmentosa, is a complex disease caused by a wide range of mutations in photoreceptors. Many of these mutations affect the expression, structure, and function of the rod visual pigment (Athanasiou et al., 2018). Because of the very high expression of opsin in the outer segments of rods, this protein plays not only a functional role, but is also critical for the proper formation of the outer segment itself. As a result, mutations affecting the expression, folding, or targeting of opsin to the rod outer segments, cause gradual degeneration of the rods. Other genes implicated in rod dysfunction and degeneration include these for phosphodiesterase (e.g., rd1, rd10; Chang et al., 2002), the CNG channels A and B subunits (channelopathies; Michalakis et al., 2018), GC, and GCAPs (Olshevskaya et al., 2002). Another diverse set of visual disorders is caused by abnormal chromophore production or supply to photoreceptors, which limits the ability to detect light and can also lead to degeneration (Ku and Pennesi, 2020). Notably, the efficiency of the visual system to produce chromophore seems to decline with age, which may result in poor rod function in dim light even in normally aging adults. It is also an early indicator for age-related macular degeneration, a devastating blinding disorder that affects the function of cones in the central retina responsible for acute vision and color discrimination (Jackson et al., 2002). Interestingly, rods and cones seem to coexist synergistically in the retina, and diseases caused by rod-specific mutations that result in rod degeneration, eventually lead to the loss of cones and central vision as well. Thus, considerable efforts are currently focused on developing methods for preserving rods even when they are not functional, as a way of protecting daytime cone-driven vision. Because the eye is a relatively accessible organ, novel therapeutic approaches for vision protection and restoration have led the field, with successful examples of gene therapy and stem cell therapy in experimental and clinical trial phases (Ovando-Roche et al., 2017).

Compared to vision, in olfactory transduction, very few mutations in transduction components are known that lead to deleterious effects. Several aspects might account for this. Mutations causing a partial reduction of olfaction might go unnoticed in the human population as very little systematic olfactory testing is done. OSNs regenerate throughout life and only have a lifespan of a few weeks. Hence any slow degeneration as those seen in photoreceptors might not manifest in that time window. In a screen of families with congenital anosmia, no potentially causative mutations were found in three main transduction proteins (G_αolf_, CNGA2, AC3), with these genes also being under purifying selection (Feldmesser et al., 2007). An interesting exception are patients suffering from retinitis pigmentosa, which is caused by mutations in the gene encoding the CNGB1 subunit expressed in both rods and OSNs. Those patients, identified because of their visual function decline, were found to be hyposmic or anosmic when tested for their olfactory ability (Charbel Issa et al., 2018). If congenital anosmia is considered to be a relatively rare and little understood condition, more known and frequently detected are specific anosmias, which manifest in the inability to detect certain odorants (Keller et al., 2007; Trimmer et al., 2019). Broadly speaking, this is the olfactory equivalent of color blindness, and is caused by known OR mutations.

Arguably, the most common causes of smell loss are events that lead to the destruction of the olfactory epithelium and/or the olfactory nerves connecting it to the central nervous system (CNS). These events include head or face trauma, inhalation of toxic chemicals, or viral infection (as SARS-CoV2), and, neurodegenerative diseases such as Alzheimer’s and Parkinson’s disease (Attems et al., 2015; Cooper et al., 2020). In the former, the origin of the smell disorder can be tracked down to the periphery, the olfactory epithelium. In the case of neurodegenerative diseases, it has been thought that olfactory dysfunction originates centrally in the CNS, but it is becoming clearer now that peripheral olfaction can be affected in these cases as well, although the respective mechanisms have not been fully elucidated.

## Future Directions

The past several decades have brought tremendous progress in our understanding of the function of photoreceptors and OSNs on the cellular and molecular levels. The development of new experimental tools, from electrophysiological recordings to genetic manipulation, advanced imaging, and genome analysis have produced a wealth of information about these exciting sensory neurons. While many questions have been answered, many more remain and will be actively investigated over the coming years. In the case of photoreceptors, one exciting area of rapidly expanding research is focused on the differences in the structure and function of foveal photoreceptors in the central part of the retina and peripheral photoreceptors (Sinha et al., 2017). Their distinct functional properties, together with their different susceptibility to degeneration in some visual disorders make it critical to understand the mechanisms that produce these differences as a mandatory step in developing successful treatments against their functional and structural loss. In addition, while gene therapy has provided some very exciting early results in treating visual disorders, a lot more work remains to be done before this approach will be ready for prime time (Garafalo et al., 2020). The recent development of retinal organoids is another exciting and rapidly growing area, which can contribute to our understanding of retinal development, gene regulation, and could provide the basis for future therapies (Ribeiro et al., 2021). Finally, the great diversity of phototransduction proteins mutations that cause visual disorders and blindness necessitates the development of gene-independent therapies for retinal disorders, whether based on common gene regulatory factors (Montana et al., 2013) or biological factors promoting photoreceptor survival (Orban et al., 2018). In vision, current research on treatments for age-related macular degeneration are at a more advanced stage (clinical trial). Several therapeutic strategies have been developed or are under investigation to address age-related macular degeneration, using antioxidative drugs, anti-inflammatory agents and inhibitors of the complement cascade, neuroprotective agents, visual cycle inhibitors, gene therapy, and cell-based therapies. Cell therapy consists of the transplantation of cells derived from embryonic or induced stem cells, or from retinal organoids, where it is possible to determine the developmental stage of the cells to transplant. One promising approach is the transplant of ectopic retinal pigmented epithelial cells (Schwartz et al., 2015; Mandai et al., 2017), in comparison to the transplant of photoreceptors, which is still lagging behind (Pearson et al., 2012; MacLaren, 2017; Ribeiro et al., 2021).

OSNs offer different kinds of complexities compared to photoreceptors, because of the much greater variety of ORs in the olfactory system, the continuous renewal of OSNs, which also necessitates their successful incorporation of OSN axons into existing olfactory circuits, and their direct exposure to the outside environment. These processes are still not well characterized and our understanding of the molecular mechanisms that regulate many of these functions, comparatively, still lags behind. As the visual system, the olfactory system is also affected by aging, causing a progressive decline of sensory abilities. The olfactory epithelium undergoes regeneration throughout the entire lifetime, but these regenerative abilities are limited. In mice, it has been shown that aging reduces the rate of regeneration of OSNs (Child et al., 2018), which results in a gradual decrease of olfaction. Different strategies against age-related smell loss have been evaluated in the past years, including the administration of growth factors 1, intranasal fibroblast growth factor-2 (Fukuda et al., 2018), activation of inositol trisphosphate receptor type 3 (IP3R3), and the neuroproliferative factor neuropeptide Y (NPY) signaling (Jia and Hegg, 2015). Given its exposure to the outside, olfaction offers itself to nasal gene delivery, which has been successfully hitherto applied to animal models of ciliopathies (McIntyre et al., 2012; Green et al., 2018). As OSNs turn over (stem), cell-based therapies might have the potential to be a more versatile and a long-term tool to restore or maintain olfactory function, potentially able to treat age-related smell loss as well as anosmia induced by viral infections, which has been surging during the current Covid-19 pandemic (Pellegrino et al., 2020).

The many parallels between photoreceptors and OSNs have helped us understand the general principles by which sensory neurons detect stimuli, amplify their signals, and adapt. Their differences have also informed us and lead us to understand how these two sensory systems deal with the specifics of the stimuli they have to transduce, the environment they operate in, and how they communicate our outside world to the brain.

## Author Contributions

FG, JR, and VK wrote the article. FG prepared the figures. All authors contributed to the article and approved the submitted version.

## Conflict of Interest

The authors declare that the research was conducted in the absence of any commercial or financial relationships that could be construed as a potential conflict of interest.

## Publisher’s Note

All claims expressed in this article are solely those of the authors and do not necessarily represent those of their affiliated organizations, or those of the publisher, the editors and the reviewers. Any product that may be evaluated in this article, or claim that may be made by its manufacturer, is not guaranteed or endorsed by the publisher.
